# Supplementary figures and images for: Joint modeling of longitudinal change in pulse rate and survival time of heart failure patients treated at Arbaminch General Hospital, Southern Ethiopia
Source: PLoS One. 2023 Mar 7;18(3):e0282637. doi: 10.1371/journal.pone.0282637 (PMC9990934; doi:10.1371/journal.pone.0282637)

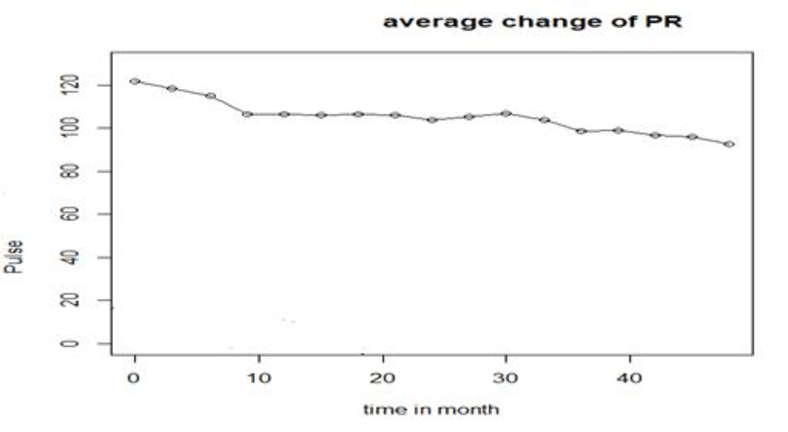

Supplement: S1 Fig — (TIF) [file pone.0282637.s001.tif]

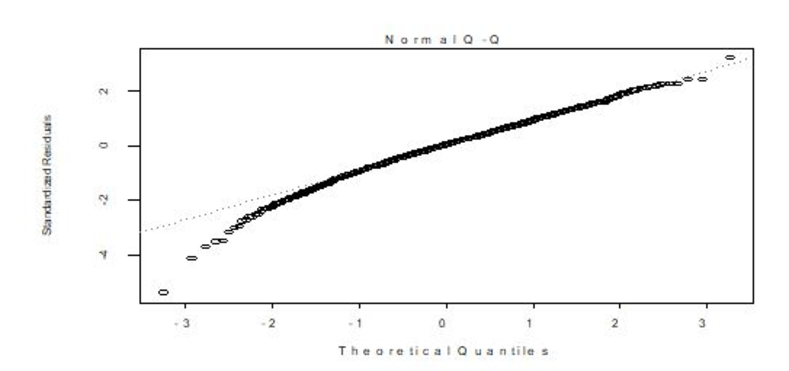

Supplement: S2 Fig — (TIF) [file pone.0282637.s002.tif]

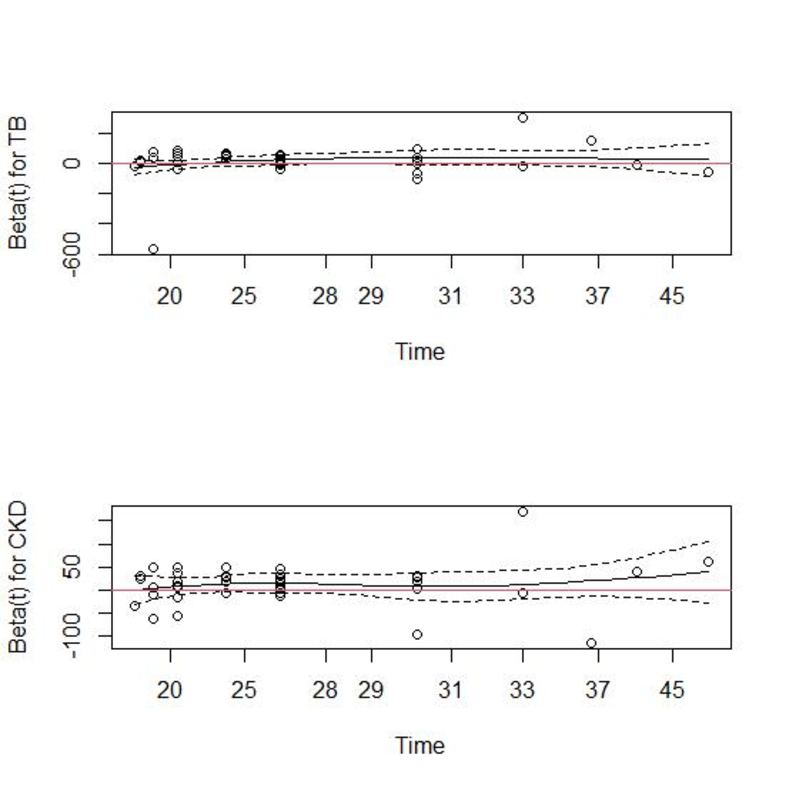

Supplement: S3 Fig — (TIF) [file pone.0282637.s003.tif]
