# Supplementary material for: Joint modeling of longitudinal change in pulse rate and survival time of heart failure patients treated at Arbaminch General Hospital, Southern Ethiopia
Source: PLoS One. 2023 Mar 7;18(3):e0282637. doi: 10.1371/journal.pone.0282637 (PMC9990934; doi:10.1371/journal.pone.0282637)
Supplement: Attachment — Submitted filename: Response to Reviewers.doc [file pone.0282637.s005.doc]

Dear academic editor and reviewers,

Thank you for your specific and helpful comments. We thank the academic editor and reviewers for the kind words on our manuscript and the points brought forward as they resulted in an improvement of the submitted manuscript. The manuscript has been improved according to the suggestions of academic editor and the reviewers. Below, we repeat the academic editor and reviewers comments, and provide our response. In the revised manuscript, the changes made to the manuscript are highlighted in yellow.

1. As suggested by the academic editor,
a. we have prepared the manuscript based on the PLOS ONE Journal requirements
b. We have amended the ethics statement to address the informed consent issue, since this study was retreospective and there was no links with individual patients, informed consent to the patient has been waived by Arba Minch General Hospital ethical committee.
c. The data set supporitng conclusions in the manusript is uploaded as supporting information file and accordingly, we have ammended data avaialablity statement in the revised manuscript.
d. We have placed ethics statement in the Methods sectionas as per your suggestions.
2. As suggested by the reviewers,
a. Thank you for making us aware of the missing categories of gender in Figure 2 which was added to the revised manuscript, we have also used PACE software to adjust for figures resolution and to change JPG file to TIFF.
b. Indeed, you are right that usually Gaussian random effects are often taken for granted in longitudinal process. We, however, checked assumption of normality of longitudinal process through normal probability plot of standardized residual from longitudinal sub model and result revealed that assumption was tenable.
c. We thankfully acknowledge your comment on the burn-in and the choice of priors, Here in our study, 20,000 iterations with different starting values were generate and inferences are made based on samples of the posterior distributions that are taken with a thinning of 10 after a burn-in of 10,000 per chain (see Fig 3 and 4). Regarding the choice of priors, we have worked in a default non-informative priors framework for the parameters which is implemented directly from the package JMbayes2 in R.
d. Thanks for your comment. Indeed, you are right that time-varying random effects could be used. We, however, decided to use random effects as a time-constant with current value parameterization in the joint model as this was also done by other studies as stated in the current manuscript. Hence, we desisted from further elaborating on this aspect in the revised manuscript.
e. We thankfully acknowledge your remark on the residual analysis, an explanatory figures for residual analysis (S1 Fig and S2 Fig) was added to the revised version of the manuscript as supplementary file, although we initially decided to not provide an additional plot as we assumed that this may distract the reader from the core of the manuscript.
f. Regarding the issues of Collinearity, We tried a slightly different specification of a model using the same data by adding and dropping a variable to inspect changes in the signs of effects on the regression coefficients (e.g. switches from positive to negative) that seem theoretically questionable if multicollinearity present.

Yours sincerely,

On behalf of the authors,

Belay Belete Anjullo
